# A rapid and efficient strategy to identify and recover biosynthetic gene clusters from soil metagenomes

**DOI:** 10.1007/s00253-022-11917-y

**Published:** 2022-04-18

**Authors:** Timo Negri, Shrikant Mantri, Angel Angelov, Silke Peter, Günther Muth, Alessandra S. Eustáquio, Nadine Ziemert

**Affiliations:** 1 Interfaculty Institute of Microbiology and Infection Medicine Tübingen (IMIT), University of Tübingen, Tübingen, Germany; 2 Interfaculty Institute for Biomedical Informatics (IBMI), University of Tübingen, Tübingen, Germany; 3 Computational Biology Laboratory, National Agri-Food Biotechnology Institute (NABI), Mohali, Punjab India; 4 NGS Competence Center Tübingen (NCCT), Institut Für Medizinische Mikrobiologie Und Hygiene, Universitätsklinikum Tübingen, Tübingen, Germany; 5 University of Illinois at Chicago, Chicago, IL USA; 6 German Centre for Infection Research (DZIF), Partner Site Tübingen, Tübingen, Germany

**Keywords:** Metagenomic DNA, Fosmid library, Nanopore sequencing, Natural products, Secondary metabolites, Single Nanopore read cluster mining (SNRCM)

## Abstract

**Abstract:**

Culture-independent metagenomic approaches offer a promising solution to the discovery of therapeutically relevant compounds such as antibiotics by enabling access to the hidden biosynthetic potential of microorganisms. These strategies, however, often entail laborious, multi-step, and time-consuming procedures to recover the biosynthetic gene clusters (BGCs) from soil metagenomes for subsequent heterologous expression. Here, we developed an efficient method we called single Nanopore read cluster mining (SNRCM), which enables the fast recovery of complete BGCs from a soil metagenome using long- and short-read sequencing. A metagenomic fosmid library of 83,700 clones was generated and sequenced using Nanopore as well as Illumina technologies. Hybrid assembled contigs of the sequenced fosmid library were subsequently analyzed to identify BGCs encoding secondary metabolites. Using SNRCM, we aligned the identified BGCs directly to Nanopore long-reads and were able to detect complete BGCs on single fosmids. This enabled us to select for and recover BGCs of interest for subsequent heterologous expression attempts. Additionally, the sequencing data of the fosmid library and its corresponding metagenomic DNA enabled us to assemble and recover a large nonribosomal peptide synthetase (NRPS) BGC from three different fosmids of our library and to directly amplify and recover a complete lasso peptide BGC from the high-quality metagenomic DNA. Overall, the strategies presented here provide a useful tool for accelerating and facilitating the identification and production of potentially interesting bioactive compounds from soil metagenomes.

**Key points:**

• *An efficient approach for the recovery of BGCs from soil metagenomes was developed to facilitate natural product discovery.*

• *A fosmid library was constructed from soil metagenomic HMW DNA and sequenced via Illumina and Nanopore.*

• *Nanopore long-reads enabled the direct identification and recovery of complete BGCs on single fosmids.*

**Supplementary Information:**

The online version contains supplementary material available at 10.1007/s00253-022-11917-y.

## Introduction

Due to the massive overuse as well as misuse of antibiotics, we currently face a worldwide health threat as a result of the increasing number of deadly infections with multi-drug resistant bacteria (Martens and Demain 2017; Church and McKillip 2021). New antimicrobial compounds are desperately needed, but for more than 30 years, no new antibiotic classes have been approved for clinical use (Church and McKillip 2021). Microbial secondary metabolites, especially the ones produced by soil microorganisms, have been a valuable source of antibiotics and also other important therapeutics such as anti-cancer drugs (Pham et al. 2019). These molecules are encoded by so-called biosynthetic gene clusters (BGCs). In each BGC, the genes necessary to build a unique compound are located next to each other within the bacterial genome (Wohlleben et al. 2016). The distinction among different classes of natural products is generally based on the mode of biosynthesis of the molecules. The most important classes in this context comprise compounds synthesized by nonribosomal peptide synthetases (NRPSs) and polyketide synthases (PKSs) as well as terpenes and ribosomally synthesized and post-translationally modified peptides (RiPPs) (Ziemert et al. 2016).

Many of these compounds were discovered from soil microorganisms by applying culture-based approaches, which rely on the isolation, culturing, and subsequent screening of the microorganisms in the laboratory for the production of bioactive compounds (Katz and Baltz 2016). While numerous compounds that are in medical use today have been discovered in this way, the culture-dependent approach soon started struggling with the issue of high rediscovery rates, leading to a decreasing number of novel compounds (Daniel 2004; Adamek et al. 2017). However, further studies revealed that soils are much richer in microbial diversity than previously anticipated and that only 1 g of soil can harbor thousands of unique bacterial species (Roesch et al. 2007). Culture-dependent approaches have so far not been able to access the majority of biosynthetic diversity present in soils and other natural environments because only a tiny proportion (approx. 1%) of bacteria can be cultured in the laboratory with the standard cultivation methods (Kellenberger 2001; Schloss and Handelsman 2003; Hug et al. 2016).

One of the attempts to solve this issue led to the development of culture-independent approaches, i.e., metagenomics, which was made possible by technological advances in sequencing, bioinformatics, and synthetic biology. It is nowadays possible to apply different sequencing approaches to soil metagenomes and yield the complete sequence of BGCs in silico (Crits-Christoph et al. 2018; Waschulin et al. 2021). However, in order to obtain the corresponding encoded compounds, the metagenomic BGCs need to be recovered and expressed in a heterologous host organism. Therefore, metagenomic DNA is generally cloned in genetic libraries such as cosmid or fosmid libraries to physically access the BGCs.

Different approaches have been applied to identify and recover BGCs from metagenomic libraries. One of these consists of screening metagenomic libraries for clones carrying BGCs via PCR using degenerate primers for biosynthetic domains such as ketosynthase (KS) domains of PKSs or adenylation (A) domains of NRPSs. Positive clones can then be recovered and further investigated for the presence of BGCs and subsequent heterologous expression experiments (Bauer et al. 2010; Amos et al. 2015). Especially larger BGCs that exceed the maximum insert size of the respective vector often reveal to be incomplete, but also smaller BGCs might only be partially captured resulting from the random cloning process. In these cases, BGC-specific primers have been used to screen the library for clones carrying the corresponding parts, enabling the assembly of the full cluster using specific cloning techniques (Hover et al. 2018; Wu et al. 2019). However, this procedure is very time consuming and more importantly requires high coverage of the full metagenome to ensure that a selected BGC is completely captured within the library. Other approaches focus on short-read shotgun sequencing of the fosmid library and subsequent identification of BGCs from the resulting contigs (Santana-Pereira et al. 2020). This approach provides more information about the presence of complete BGCs within the library for potential heterologous expression. However, it also does not give direct information on the distribution of the BGCs over different clones, which makes recovery of complete BGCs still a time-consuming labor-intensive process.

Here, we developed a method we called single Nanopore read cluster mining (SNRCM), which uses short- and long-read sequencing data of a fosmid library to accelerate the recovery of complete secondary metabolite BGCs. This approach enabled us to use Nanopore long-reads for the identification of complete BGCs on a single fosmid without prior isolation and analysis of candidate clones. Furthermore, using the sequencing data of the corresponding metagenomic DNA, we were able to directly amplify a complete lasso peptide BGC from metagenomic DNA, as well as assemble a larger NRPS BGC spanning over different fosmids into a complete cluster.

## Materials and methods

### Soil sampling, isolation, and sequencing of metagenomic HMW DNA

The A horizon of the soil type cambisol was sampled from the Schönbuch Forest nature reserve (close to Tübingen, Germany) in November 2016. Different metagenomic HMW DNA isolation protocols were applied for subsequent Nanopore and Illumina sequencing of the DNA as described in detail in our previous study (Mantri et al. 2021). The HMW DNA used for fosmid library generation was isolated following two methods of our previous study, which are summarized in the following: a small part of the library (approx. 8000 clones) was generated from HMW DNA isolated using the isolation method “for Nanopore sequencing run 1.” Briefly, this method followed a published isolation protocol (Brady 2007) with an additional step to increase the DNA purity. The protocol is based on chemical lysis using a heated lysis buffer (70 °C) to extract metagenomic DNA from a 250 g fine soil sample. After precipitation with isopropanol, the DNA was purified and size selected using a large agarose gel. For that purpose, the resuspended DNA was loaded into several large wells, followed by electrophoresis at 20 V overnight. Subsequently, a left and right part of the gel each containing the Lambda DNA/HindIII ladder and a small part of a well were cut, stained with ethidium bromide and visualized. The ladder served as reference to mark the location of HMW DNA within the gel. After reassembly of the gel, the marks were used to cut gel slices from the unstained gel containing the HMW DNA. The slices were transferred to dialysis tubes, followed by electroelution of the HMW DNA contained within the gel slices into the dialysis tubes. As an additional step to the published protocol, the dialysis tubes were incubated overnight in 0.5 × Tris–EDTA (TE) buffer. Finally, the DNA was concentrated using a centrifugal concentrator (Amicon, MWCO 30,000) as described by Brady. The main part of the fosmid library (75,700 clones) was generated from HMW DNA isolated using the isolation method “for Nanopore sequencing run 2” described in our previous study. Briefly, this method followed a published isolation protocol (Verma et al. 2017) with several modifications to optimize DNA yield and purity. The protocol is based on a combination of enzymatic (37 °C) and subsequent chemical lysis (65 °C) to extract metagenomic HMW DNA from 6 fine soil samples, each of 5 g. The extract was first purified with chloroform/isoamyl alcohol, followed by precipitation of the DNA with a 0.1 volume of 3 M sodium acetate and a 0.4 volume of 30% poly ethylene glycol (PEG-8000). The DNA was resuspended in TE buffer, and, as an additional step to the published protocol, 1 μl of RNase I was added, followed by incubation for 30 min at 37 °C. Subsequently, the DNA was extracted with chloroform/isoamyl alcohol. As another modification to the protocol, in the following step, the DNA was not only precipitated with isopropanol as described but also with a 0.1 volume of 5 M sodium acetate. The protocol of Verma et al. was completed by washing the pelleted DNA with a sodium chloride solution and subsequently with 70% ethanol, followed by resuspension in TE buffer. The obtained DNA was further purified and size selected using a large agarose gel as described above. The size of the resulting DNA was analyzed by gel electrophoresis on a 1% agarose gel and visualized using a gel imaging system (Nippon Genetics Europe). This DNA was also used for the amplification of metagenomic BGCs. Concentration and absorbance ratios were determined using the Nanodrop 2000c spectrophotometer (Thermo Scientific).

### Generation of a metagenomic fosmid library and subsequent Illumina and Nanopore sequencing

Isolated metagenomic HMW DNA was directly used for the generation of a metagenomic fosmid library using the CopyControl Fosmid Library Production Kit with pCC1FOS Vector (Lucigen) following the manufactures instructions with the following modifications: Since the DNA was already size selected during the isolation procedure, it had the appropriate size and did not need any shearing or further size selection after end-repair. The ligation reaction was scaled up to 20 μl for each packaging reaction. Ligation was performed overnight at 16 °C. Clones were stored in pools of 2000 clones following storage method C of the manual. Aliquots of each pool were pooled and used to inoculate 100 ml of lysogeny broth (LB) medium supplemented with 12.5 μg/mL chloramphenicol (CHL) and 1 × CopyControl Fosmid Autoinduction Solution and grown overnight at 37 °C with shaking. After the cells were pelleted, the following steps were conducted by the NGS Competence Center Tübingen (NCCT): Fosmids were isolated from the pelleted cells using the ZymoPURE Plasmid Miniprep kit (Zymo Research). Residual genomic DNA was digested using Exonuclease V (NEB), followed by purification of the fosmids using the Genomic DNA Clean & Concentrator-10 kit (Zymo Research). For Illumina sequencing, fosmids were sheared using a Covaris M220 Focused-ultrasonicator device. Library preparation was conducted using the TruSeq DNA PCR-Free kit (Illumina) and paired-end sequencing (2 × 150 bp) was performed on a NextSeq 550 system using a NextSeq Mid Output flow cell (300 cycles). For Nanopore sequencing, library preparation was performed using the Rapid Barcoding Kit (SQK-RBK004). Sequencing was performed on a MinION device using a MinION flow cell (version R9.4.1).

### Hybrid assembly of the fosmid library sequencing data and subsequent identification of complete natural product BGCs

SPAdes version 3.11.1 (Antipov et al. 2016) was used for hybrid assembly of Illumina and Nanopore data. “meta” flag enabling the assembly of metagenomic datasets was used during this assembly, which was performed on deNBI cloud virtual machine having 36 CPU cores and 1.5 Terabytes of RAM. Custom perl script was used for filtering the hybrid contigs based on their length. BGCs were identified using antiSMASH version 5 (Blin et al. 2019). BGCs on contigs annotated as being complete were aligned with Nanopore reads (SNRCM approach) using BLAST analysis (Zhang et al. 2000) to identify clones carrying complete BGCs on a single fosmid.

### Recovery of clones of interest from pools of 2000 clones using serial dilution PCR

Specific primers for each clone of interest were designed using Geneious version 9.1.8. Clones of interest were recovered from pools of 2000 clones by applying a serial dilution PCR method (Owen et al. 2015) with the following specifics: The respective positive *E. coli* pool of interest was grown overnight in LB containing 12.5 μg/ml CHL. The pool was diluted with LB medium to an OD_600_ of 0.25 × 10^−5^ (≈ 2000 cells/ml). Sixteen to 32 glass culture tubes containing 5 ml LB supplemented with CHL and 1 × autoinduction solution were inoculated each with 100 μl (≈ 200 cells) of the pool dilution and grown overnight. Plasmids of the pool dilutions were isolated by alkaline lysis and screened for the target gene via PCR. The positive pool was diluted to approx. 200 cells/ml (OD_600_ of 0.25 × 10^−6^) and 200 μl (≈ 40 cells); each was used to inoculate 16–32 glass culture tubes containing 5 ml LB (CHL, autoinduction solution) and grown overnight. Plasmids of the overnight cultures were isolated and screened for the target gene via PCR. The positive pool was diluted until a dilution factor of 10^–7^ was reached and 100 μl were plated on LB agar (CHL 12.5 μg/ml). Single colonies were grown overnight in 5 ml LB (CHL, autoinduction solution) and the respective plasmids were isolated and screened via PCR for the fosmid of interest.

### Identification of lasso peptide-specific genes

The minimal set of the lasso peptide biosynthesis gene homologues A, B and C for the metagenomic BGCs 40.1 and 482.1 as well as genes A, B, C, and D for BGC 44.1 were identified using Blastx and antiSMASH analysis. Lasso peptide A genes were identified either by antiSMASH annotation (BGC 44.1) or manual inspection of the translated nucleotide sequence of candidate genes for the characteristics common for lasso peptide formation (BGC 482.1). The B1 genes were identified by comparing the Blastx analysis results, gene sizes, and antiSMASH annotations of known lasso peptide B1 genes from the MIBiG database (Kautsar et al. 2019) with the results for genes within the metagenomic lasso peptide BGCs. The B2 genes were directly identified by Blastx analysis. C genes were identified by antiSMASH, generally annotating C genes as “Asn synthase.” The D gene often codes for a transporter of the ABC type and was identified in BGC 44.1 via Blastx and antiSMASH analysis.

### Amplification and cloning of a lasso peptide BGC from metagenomic HMW DNA

The lasso peptide BGC 44.1 that was detected on hybrid assembled contigs (Online Resource 3) derived from the metagenome sequencing data of our previous study (Mantri et al. 2021) was used as a reference sequence to design the specific primers Cl_44_SalI_fw GTCGACCCTCCGTCGCAGAGCTGTAT (SalI recognition site underlined) and Cl_44_SacI_rv GAGCTCAAGATGTTCCTGACCTGCGG (SacI recognition site underlined). Using this primer pair as well as the Q5 high-fidelity DNA polymerase kit (NEB), the lasso peptide-specific genes A, B1, B2, and C of BGC 44.1 were amplified via PCR. Five 25 μl reactions with varying metagenomic DNA template amounts (114 ng, 54 ng, 30 ng, 15 ng, and 3 ng) and the following reaction mixture were performed: 5 μl of 5 × Q5 reaction buffer, 5 μl of 5 × Q5 High GC Enhancer, 0.5 μl of 10 μM forward/reverse primer, 0.5 μl of 10 mM deoxynucleotide triphosphates (dNTPs), 3 μl of template DNA, 0.25 μl of Q5 high-fidelity DNA polymerase, and 10.25 μl of nuclease-free water. Thermocycling conditions were as follows: 98 °C for 30 s, 30 cycles of 98 °C for 10 s, 67 °C for 30 s, 72 °C for 70 s, and a final step with 72 °C for 2 min. Five microliter of each reaction was analyzed on a 1% agarose gel and the remainder of each reaction (20 μl) was pooled. Pooled BGC 44.1 amplicons were purified using the Genomic DNA Clean & Concentrator-10 kit (Zymo Research) following the manufacturer’s instructions. Purified PCR products were digested with SacI (Thermo Scientific) overnight and subsequently purified. Purified SacI-digested PCR products were then digested with SalI (Thermo Scientific) overnight and subsequently purified again. Purified SacI/SalI-digested PCR products were ligated into the equally digested and purified expression vector pSK019 (Kunakom and Eustáquio 2020) using the T4 DNA Ligase (Thermo Fisher Scientific) and following the manufacturer’s instructions, which resulted in the generation of pSK019_44.1. Five microliter of the ligation mixture was used to transform 100 μl of *E. coli* DH10ß cells via electroporation (Bio-Rad MicroPulser Electroporator). Transformed cells were plated on LB agar containing kanamycin (50 μg/ml) and incubated overnight. Six transformants were picked and grown in LB kanamycin (50 μg/ml) overnight followed by plasmid isolation using alkaline lysis. Isolated plasmids were screened for the presence of BGC 44.1 by performing PCR with the above described conditions and primers. Positive plasmids were Sanger sequenced using sequencing primers that generate overlapping sequences. The corresponding D gene was amplified from metagenomic DNA by PCR as described above, using primers Cl44_D_gene_OV_fw GGTCAGGAACATCTTGAGCCCGAACAGCAATGACAGAAC and Cl44_D_gene_OV_rv CATGATTACGAATTCGAGCGCCGCCTTCTTGCAATTAA with overhangs (underlined) that allowed assembly of the product with pSK019_44.1. PCR conditions were as described above with an adjusted annealing temperature of 66 °C and elongation time of 40 s. Five microliter of each reaction was analyzed on a 1% agarose gel, and the remainder of each reaction (20 μl) was pooled. Pooled PCR products were purified using the Genomic DNA Clean & Concentrator-10 kit. pSK019_44.1 was linearized by digestion with Ecl136II (Thermo Scientific) overnight and subsequently purified. Purified D gene with overhangs and purified linearized pSK019_44.1 were assembled using the NEBuilder HiFi DNA Assembly Master Mix (NEB), which resulted in the construction of pSK019_44.1_D. The construct was transferred to *E. coli* DH10ß, and positive clones were verified via PCR using the same primers that were used for D gene amplification. A positive plasmid was Sanger sequenced using sequencing primers that generate sequences with overlap, thereby covering BGC 44.1 lasso peptide-specific genes A, B1, B2, and C as well as genes D1 and D2. Generated Sanger sequences were aligned with the reference sequence using Geneious.

### Culture conditions, extraction method, and analysis of extracts of lasso peptide heterologous expression experiments

Glass culture tubes containing 5 ml of LB medium (CHL) were inoculated using glycerol stocks of three *E. coli* DH10ß clones carrying lasso peptide BGCs 40.1 and 482.1 on fosmids, respectively, as well as a strain carrying the empty pCC1FOS vector (negative control), and the cultures were grown at 37 °C with shaking overnight (ON). Erlenmeyer flasks containing 100 ml of M9 minimal media (Zhu et al. 2016) supplemented with CHL were inoculated with 1 ml of each ON culture, and the cultures were grown for three days at 37 °C with shaking, followed by extraction.

Precultures, that is, ON cultures, of three *E. coli* DH10ß clones carrying lasso peptide BGC 44.1 cloned into pSK019 as well a strain carrying the empty pSK019 vector (negative control) were prepared as described above using 50 μg/ml kanamycin (KAN) instead of CHL for selection of the plasmid. Erlenmeyer flasks containing 100 ml of M9 minimal medium (4 g/l glycerol instead of glucose as carbon source) supplemented with 50 μg/ml KAN and 100 mM L-Arabinose for induction of the PBAD promoter were inoculated with 1 ml of each ON culture. The cultures were grown for 3 days at 37 °C with shaking and were subsequently extracted.

The lasso peptide BGC 44.1 carrying plasmid pSK019_44.1_D as well as the empty pSK019 vector were transferred to *Burkholderia* sp. FERM BP-3421 (International Patent Organism Depository at the National Institute of Advanced Industrial Science and Technology, Tsukuba, Japan) via electroporation following a published protocol (Kunakom and Eustáquio 2020). Three BGC carrying clones as well as the strain carrying the empty vector (negative control) were inoculated in 5 ml LB medium supplemented with 500 μg/ml kanamycin (KAN500), and the cultures were grown for 2 days at 30 °C with shaking. Erlenmeyer flasks containing 100 ml of M9 minimal medium (4 g/l glycerol instead of glucose as carbon source) supplemented with KAN500 and 100 mM L-Arabinose for induction of the PBAD promoter were each inoculated with 1 ml of the precultures. The cultures were grown for 3 days at 30 °C with shaking and were subsequently extracted.

For lasso peptide extraction from the 100 ml cultures, the media was separated from the cells by centrifugation (20 min, 4000 rpm). Cell pellets were frozen at − 80 °C and thawed again, followed by extraction with 50 ml of methanol (MeOH) and shaking overnight. The extracts were centrifuged (10 min, 4000 rpm), and the MeOH was subsequently transferred to round-bottom flasks. Using a rotary evaporator the extracts were dried at 37 °C and applying reduced pressure.

For lasso peptide extraction from the culture media, XAD-16 resin (Amberlite) was used. Fifteen milliliter of a XAD-16/water suspension was added per 100 ml of media and incubated for 1 h with shaking. Subsequently, the XAD-16 was separated from the media and washed with water, followed by extraction with 100 ml of MeOH and shaking for 30 min. The MeOH was transferred to round-bottom flasks and the extracts were dried at 37 °C using a rotary evaporator and applying reduced pressure. Dried extracts were dissolved in 50% MeOH and subsequently analyzed using a HPLC 1260 Infinity device (Agilent Technologies) coupled to an InfinityLab LC/MSD mass spectrometry device (Agilent Technologies). HPLC was performed using a Kinetex 5 μm C18 100 Å LC column (100 × 4.6 mm) and applying a gradient (10–100%) of acetonitrile (0.1% formic acid) in water (0.1% formic acid) for 20 min at a flow rate of 1 ml/min. Mass spectrometry was performed using positive ion mode configuration with a mass range of 100–2000 Da. Analysis of the generated data was conducted using the data analysis tool of the LC/MSD ChemStation software (Agilent Technologies).

### TAR cloning of a NRPS BGC distributed over 3 different fosmids

The NRPS BGC to be TAR cloned from the fosmid library was detected on a 138.907 bp contig derived from the hybrid assembly of the corresponding metagenome sequencing by antiSMASH analysis and served as a reference sequence for the TAR cloning experiment. Cluster parts were detected on different hybrid assembled contigs derived from the fosmid library sequencing. In order to assemble the BGC to a complete cluster, the fosmid library was screened for clones carrying overlapping parts of NRPS BGC 76.1 using primers specific for the left (fw: GGTGACCCGACAATTCCCAT, rv: TCACCGTGAGCTTCAGTGAC), middle (fw: CGGATTCCTGTGCTCTGGTT, rv: TTGCCAATTAGACCGGACCC), and right part (fw: CAAAGACACGCAAGCAGCTT, rv: TCTTTGAGCAGGGTTCCGTC) of the BGC via PCR, and positive clones were isolated by serial dilution PCR. The sequence parts carried by each clone were determined by end sequencing from both sides of each fosmid and aligning the sequences with the 138.907 bp contig using Geneious. The fosmids of three clones covering the complete BGC with overlapping parts of the cluster were isolated and each digested with one of the three restriction enzymes DraI, Eco105I, or PsiI (Thermo Scientific) in order to linearize the fosmids and release overlapping parts of the BGC necessary for assembly. The digested fosmids were subsequently purified by phenol/chloroform extraction. TAR cloning was performed following a published protocol with a few modifications (Zhang et al. 2019). Briefly, a 144 bp dsDNA fragment containing the cluster-specific 50 bp hooks was synthesized (IDT). The synthesized fragment was amplified by PCR and subsequently assembled with the XhoI/NdeI (Thermo Scientific) digested pCAP03 vector by Gibson Assembly (NEB). The assembled vector was cloned in *E. coli* DH10ß cells and subsequently isolated by alkaline lysis. The PmeI (Thermo Scientific) digested vector was then used together with the three digested fosmids for TAR cloning in yeast. Yeast colonies were screened by colony PCR using primers targeting the left, middle, and right part of the BGC. The plasmid of positive colonies was isolated using the Zymoprep Yeast Plasmid Miniprep I kit (Zymo Research) and transferred to *E. coli* DH10ß via electroporation. The plasmid was subsequently isolated from *E. coli* by alkaline lysis, and residual genomic DNA contamination was removed using Exonuclease V (NEB), followed by purification of the plasmids using the Genomic DNA Clean & Concentrator-10 kit. Purified plasmids were digested with *Smi*I (Thermo Scientific) overnight in order to generate two linear DNA fragments for Nanopore sequencing that were subsequently purified again. Sequencing library preparation was conducted using the Ligation Sequencing Kit SQK-LSK109 with Native Barcoding Expansion (EXP-NBD104). Sequencing was performed by the NCCT on a PromethION device using a PromethION flow cell (version R9.4.1). Nanopore reads were size filtered in order to select for the sizes of the two fragments and aligned with the reference sequences generated from the metagenome sequencing using Tablet (version 1.19.09.03).

## Results

### Isolation of high-quality HMW metagenomic DNA from soil

**Fig. 1:**
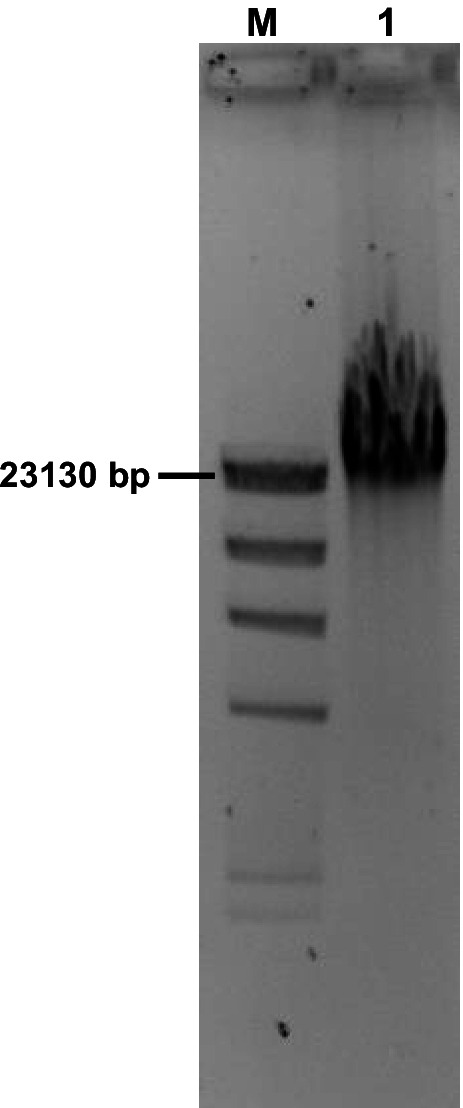
Gel electrophoresis of metagenomic HMW DNA on a 1% agarose gel. Lane M, Lambda DNA/HindIII Marker; lane 1, metagenomic HMW DNA isolated from a soil sample of the Schönbuch forest

A prerequisite for the development of an efficient approach for the fast discovery and heterologous expression of novel BGCs from soil metagenomes is the isolation of high-quality HMW metagenomic DNA. In this study in particular, the metagenomic DNA needed to be of sufficient quality for the downstream applications we used such as long- and short-read sequencing, fosmid library generation and direct amplification of complete BGCs via PCR. We used our optimized metagenomic DNA isolation protocol (Mantri et al. 2021) to isolate high-quality HMW metagenomic DNA from a soil sample of the Schönbuch forest (Germany). Analysis of the DNA on a gel revealed an intense band migrating above the lambda DNA/HindIII 23 kb band with almost no smear below, which confirmed that the DNA was of a high molecular weight and showed minimal shearing (Fig. 1). An absorbance 260/280 ratio of 1.86 and a 260/230 ratio of 1.67 further confirmed the high purity of the isolated DNA.

### Generation and sequencing of a metagenomic fosmid library

**Fig. 2:**
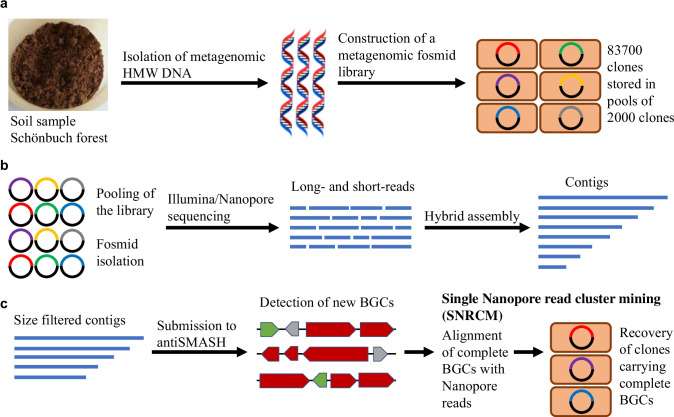
Workflow for capturing metagenomic BGCs ready for heterologous expression. **a** Isolation of high quality HMW DNA from soil and subsequent construction of a metagenomic fosmid library consisting of approx. 83,700 clones stored in pools of 2000 clones. **b** Pooling of the library and isolation of fosmids for subsequent Illumina/Nanopore sequencing. Hybrid assembly of short- and long-reads. **c** Size filtering of contigs greater than 40 kb and subsequent submission to antiSMASH for BGC detection. Alignment of complete BGCs with Nanopore reads for identification of fosmids harboring a complete BGC. Recovery of positive clones by serial dilution PCR

The isolated metagenomic DNA was directly used for generating a fosmid library of more than 83,000 clones (Fig. 2a). The entire fosmid library was sequenced using Illumina and Nanopore technologies. Contigs were assembled using a hybrid assembly approach with both short- and long-reads (Fig. 2b) resulting in nearly 16 thousand contigs greater than 25 kb (summary of sequence data in Supplemental Table S1).

At the same time, the corresponding soil metagenomic DNA was sequenced using Illumina and Nanopore and contigs were generated using the same metaSPAdes-based hybrid assembly approach, which was published in our previous study (Mantri et al. 2021).

### AntiSMASH analysis reveals the presence of a large number of new natural product BGCs within the fosmid library

In order to obtain a first overview of the total amount of the BGCs, hybrid assembled contigs greater than 1 kb were analyzed with antiSMASH (Blin et al. 2019), which revealed the presence of 2019 BGC regions captured within the library.

Closer inspection of the detected BGCs within the fosmid library revealed that the majority consisted of incomplete clusters, which are often annotated by antiSMASH as “region on contig edge.” Since our approach aimed at recovering complete BGCs ready for heterologous expression, we filtered for contigs greater than 40 kb (Fig. 2c). The antiSMASH analysis identified 100 BGCs (Online Resource 1) of which 98 showed no significant similarity to any characterized BGC within the MIBiG database (Kautsar et al. 2019). Thirty-four of these were annotated as being complete, and the encoded compounds were predicted to belong to diverse natural product classes: five type 3 PKSs, two NRPSs, nine terpenes, seven lasso peptides, five bacteriocins, and one of each: a heterocyst glycolipid synthase-like PKS (hglE-KS), an aryl polyene, a lanthipeptide, an indole/terpene, a betalactone, and a linear azol(in)e-containing peptide (LAP).

### Identification and recovery of completely captured BGCs on single fosmids

Each of the 34 complete BGCs on hybrid assembled contigs could potentially have been distributed over different fosmids of the sequenced library, thus requiring multiple steps for complete BGC recovery. To select complete BGCs captured on a single fosmid, we developed a fast and more efficient recovery approach and called it single Nanopore read cluster mining (SNRCM): We used the generated high quality long-read Nanopore data and aligned the 34 complete BGCs directly with the single Nanopore reads (Fig. 2c) (Online Resource 2). 15 BGCs aligned completely with a Nanopore read. As every Nanopore read is derived from one fosmid DNA molecule, these 15 BGCs must have been captured completely on a single fosmid. These BGCs were predicted to encode lasso peptides (3 BGCs), bacteriocins (5), betalactones (1), LAPs (1), lanthipeptides (1), and terpenes (4).

**Fig. 3:**
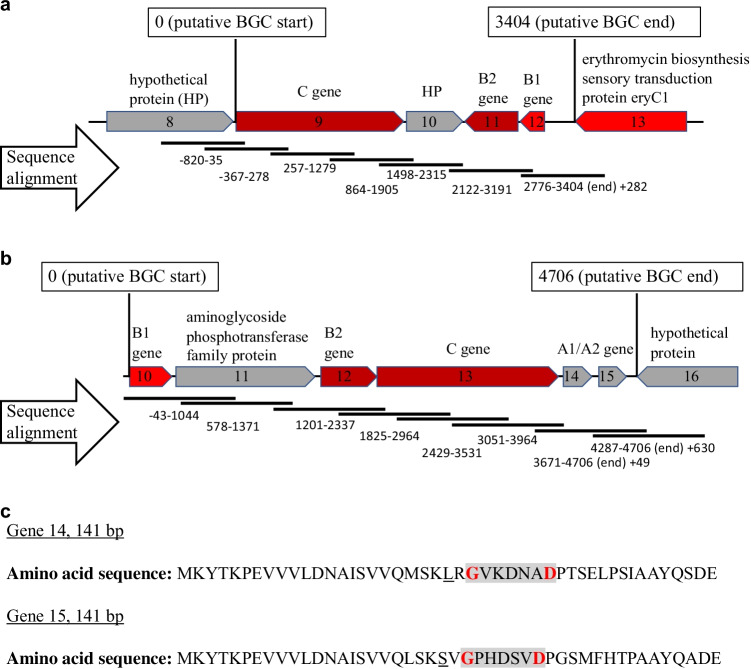
Sequencing confirmation for lasso peptide BGCs 40.1 and 482.1. Sequencing of lasso peptide BGC 40.1 (**a**) and 482.1 (**b**) using specific sequencing primers that generate sequences with overlap. Numbers of the alignment refer to the start and end point of each generated sequence that matches the reference sequence. Gene annotation via bioinformatics analysis as described. Translated nucleotide sequences of genes 14 and 15 of lasso peptide BGC 482.1 (**c**). Most suitable amino acid candidates for ring formation in bold and red. Amino acids building the putative ring with grey shade. Amino acids similar in size to a commonly found threonine at position -2 relative to the core peptide underlined

As a proof of concept, two lasso peptide encoding BGCs (40.1 and 482.1) were chosen, and the corresponding fosmids were isolated. The BGCs were analyzed for completeness by checking for the presence of the minimal set of the necessary lasso peptide biosynthesis gene homologues A, B, and C. For both BGCs, the B2 and C genes could be identified using Blastx and antiSMASH analysis (Table 1, Supplemental Table S2). The putative B1 genes were identified by comparing the Blastx analysis results, gene sizes, and antiSMASH annotations to known lasso peptide B1 genes (MIBiG) (Table 1, Supplemental Tables S2 and S3). The precursor peptides in BGC 482.1 (A genes) had to be manually annotated as it is often the case for lasso peptide encoding BGCs due to the small size of the gene. BGC 482.1 revealed two putative A genes. These genes not only showed a similar size to known A genes, but, more importantly, their translated amino acid sequences (Fig. 3c) revealed characteristics common to a lasso peptide (Maksimov et al. 2012). These included (i) the presence of a glycine in appropriate distance to an aspartate for ring formation and (ii) appropriate length of the amino acid sequence predicted to form the tail of the lasso peptide, i.e., those amino acids that were located right to the ring forming amino acids within the sequence. The amino acids on the left side of the ring forming glycine would be those cleaved from the precursor peptide. Although a threonine is commonly found at position -2 relative to the core peptide, the threonine can be replaced with amino acids similar in size (Pan et al. 2012) such as the leucine and serine predicted here. The putative lasso peptide-specific genes on both isolated fosmids were resequenced to confirm the recovery of two complete clusters each captured on a single fosmid (Fig. 3a and b). Multiple candidate precursor genes were detected within BGC 40.1 that fulfilled the abovementioned characteristics; however, no specific candidate was singled out as the most promising precursor.

**Table 1:** Identification of putative lasso peptide genes of BGCs 40.1 and 482.1 using Blastx results, gene sizes and antiSMASH annotations. Blastx results, gene sizes, and antiSMASH annotations with high similarities to known B1 genes highlighted in bold

Gene	Blastx results	Gene size (bp)	antiSMASH annotation	Putative lasso peptide gene
Metagenomic lasso peptide BGC 40.1				
8	Hypothetical protein [*Acidobacteriia bacterium*]	975	-	-
9	Hypothetical protein [*Acidobacteriia bacterium*]	1905	Asn_synthase	C gene
10	Hypothetical protein [*Betaproteobacteria bacterium*]	432	-	-
11	Lasso peptide biosynthesis B2 protein [*Acidobacteriia bacterium*]	423	PF13471	B2 gene
12	**PqqD** **family** **peptide** **modification** **chaperone** [*Acidobacteriia bacterium*]	**294**	**PF05402**	B1 gene
13	Erythromycin biosynthesis sensory transduction protein eryC1 [*Acidobacteria bacterium*]	1116	DegT_DnrJ_EryC1	-
Metagenomic lasso peptide BGC 482.1				
10.1	Hypothetical protein DMG37_22385 [*Acidobacteria bacterium*]	**276**	**PF05402**	B1 gene
10.2	**PqqD** **family** **protein** [*Acidobacteriia bacterium*]			
11	Aminoglycoside phosphotransferase family protein [*Acidobacteriia bacterium*]	1404	-	Kinase
12	Lasso peptide biosynthesis B2 protein [*Acidobacteria bacterium*]	375	PF13471	B2 gene
13	Hypothetical protein [*Acidobacteriia bacterium*]	1929	Asn_synthase	C gene
14	Hypothetical protein [*Acidobacteria bacterium*]	141	-	A1 gene
15	Hypothetical protein [*Acidobacteria bacterium*]	141	-	A2 gene
16	Hypothetical protein DMG78_32005 [*Acidobacteria bacterium*]	2118	-	-

Overall, this strategy allowed us to isolate single fosmids that harbored complete BGCs and were ready for downstream heterologous expression in *E. coli* or other established hosts upon introduction of the required genetic elements for maintenance and transfer into the backbone of the fosmids.

We attempted heterologous expression of the two isolated lasso peptide BGCs in *E. coli* by culturing them in M9 minimal media, followed by separate extraction of the cell pellets and culture media. However, analysis of the extracts via high-performance liquid chromatography-mass spectrometry (HPLC–MS) did not lead to the detection of the expected lasso peptides.

### Using HMW metagenomic DNA for direct amplification of BGCs via PCR and cloning into expression vectors

**Fig. 4:**
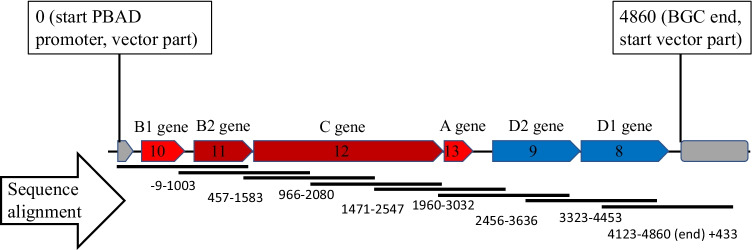
Sequencing confirmation for lasso peptide BGC 44.1 cloned into an expression vector. Sequencing of lasso peptide BGC 44.1 using specific sequencing primers that generate sequences with overlap. Numbers of the alignment refer to the start and end point of each generated sequence that matches the reference sequence. Vector derived PBAD promoter determined as starting point of the reference sequence. Gene annotation via bioinformatics analysis as described

The isolation of HMW metagenomic DNA from soil samples also allows the direct amplification of complete BGCs via PCR and subsequent cloning into expression vectors independent of genomic libraries. To demonstrate this, we chose the lasso peptide BGC 44.1 detected on a contig derived from the soil metagenome sequencing (Online Resource 3). The putative lasso peptide specific genes A, B1, B2, C, and D were identified and analyzed using the bioinformatics methods as described before (Table 2, Supplemental Table S4). Using specific primers with restriction site overhangs (SacI, SalI) compatible for subsequent cloning, the genes A, B1, B2, and C, all showing the same orientation, were amplified in a single PCR (3034 bp). Subsequently, the amplified cluster was ligated into an expression vector. In a second step, the translationally coupled genes D1 and D2 were similarly amplified in a single PCR using specific primers with overhangs that allowed the subsequent introduction of the amplified D genes downstream of the other lasso peptide-specific genes via Gibson Assembly. Sequencing of the final construct confirmed the successful cloning of the cluster (Fig. 4) and proved the feasibility of directly amplifying small BGCs from metagenomic DNA for subsequent cloning into expression vectors.

**Table 2:** Identification of putative lasso peptide genes of BGC 44.1 using Blastx results, gene sizes, and antiSMASH annotations. Blastx results, gene sizes, and antiSMASH annotations with high similarities to known B1 genes highlighted in bold

Metagenomic lasso peptide BGC 44.1				
Gene	Blastx results	Gene size (bp)	antiSMASH annotation	Putative lasso peptide gene
8	ABC transporter permease [*Acidobacteriia bacterium*]	822	-	D1 gene
9	ABC transporter ATP-binding protein [*Acidobacteriia bacterium*]	810	ABC transporter ATP-binding protein	D2 gene
10	**PqqD** **family** **peptide** **modification** **chaperone** [*Acidobacteriia bacterium*]	**303**	**PF05402**	B1 gene
11	Lasso peptide biosynthesis B2 protein [*Acidobacteriia bacterium*]	471	PF13471	B2 gene
12	Asparagine synthetase B [*Acidobacteriia bacterium*]	1863	Asn_synthase	C gene
13	Hypothetical protein DMG36_15005 [*Acidobacteria bacterium*]	150	Predicted lasso peptide	A gene

Heterologous expression of the cloned lasso peptide BGC was attempted in two heterologous hosts (*E. coli* and *Burkholderia* sp. FERM BP-3421). However, no lasso peptide was detected upon HPLC–MS analysis of the extracts.

### Assembly of a large NRPS cluster distributed over 3 different fosmids via transformation-associated recombination (TAR) cloning

**Fig. 5:**
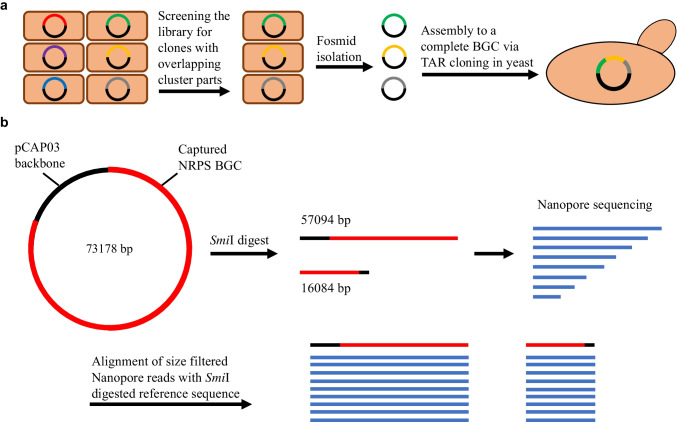
Workflow for the assembly of a large NRPS BGC from cluster parts on different fosmids via TAR cloning. **a** Screening of the fosmid library for clones carrying fosmids with overlapping BGC parts and subsequent isolation of the respective clones. Fosmid isolation and assembly of the cluster parts into a complete cluster via TAR cloning in yeast. **b** *Smi*I digest of the assembled NRPS BGC generates two fragments for subsequent Nanopore sequencing. Size filtering of the generated reads and alignment with *Smi*I digested reference sequence

Using both sequence information of a generated metagenomic fosmid library as well as its corresponding metagenomic DNA not only allows for the recovery of small BGC classes such as RiPPs but also for other classes that are typically larger in size even when library size is limited. We demonstrated this by recovering an approximately 58 kb NRPS BGC from the fosmid library. The complete NRPS BGC 76.1 was detected on a 139 kb contig derived from the direct soil metagenome sequencing (Online Resource 3), while only parts of the cluster were detected on different fosmid library contigs. Specific primers were designed to screen the fosmid library for those clones that harbored overlapping BGC parts and together covered the complete BGC (Fig. 5a). Three clones and the respective fosmids were isolated, and the different parts of the BGC were assembled to a complete cluster using TAR cloning (Zhang et al. 2019) (Fig. 5a). The final plasmid was cut into two fragments of approximately 57 kb and 16 kb by restriction digestion and sequenced via Nanopore. The Nanopore reads were filtered for the respective fragment sizes and aligned with the reference sequence derived from the metagenome sequencing (Fig. 5b) (Online Resource 4), which proved the correct assembly of the complete NRPS BGC. Following this approach, TAR cloned metagenomic BGCs can directly be transferred to compatible heterologous hosts such as *Streptomyces* species as different TAR vectors contain the respective necessary genetic elements for this purpose.

Our results show that the generation of a metagenomic fosmid library from high-quality HMW DNA coupled with short- and long-read sequencing of both enables the fast and efficient recovery of metagenomic BGCs ready for heterologous expression.

## Discussion

Multiple studies have revealed the huge biosynthetic diversity of soil metagenomes potentially coding for therapeutically relevant compounds (Charlop-Powers et al. 2015; Mantri et al. 2021) and further showed that the biosynthetic diversity can be successfully captured within corresponding metagenomic libraries (Parsley et al. 2011; Reddy et al. 2012; Santana-Pereira et al. 2020). The isolation of high-quality HMW DNA is not only a crucial step for library generation, but it is also the basis for downstream applications associated with the identification and recovery of complete BGCs such as NGS sequencing, PCR, and DNA cloning. This first step poses a major challenge especially for soils as they harbor various impurities such as humic acids, which are often copurified with DNA (Amorim et al. 2008; Sar et al. 2018) and can interfere with the aforementioned downstream applications (Nair et al. 2014; Verma et al. 2017). The HMW metagenomic DNA we isolated proved to be suitable for yielding high-quality short- and long-read sequencing data, amplifying complete BGCs via PCR and generating a high-quality metagenomic fosmid library.

Metagenomic libraries are often the starting point for the recovery and heterologous expression of BGCs (Katz et al. 2016). Screening of a metagenomic library for the presence of clones that carry potentially interesting BGCs is often a time-consuming multi-step process. Here we show that combining short- and long-read sequencing of our metagenomic fosmid library and applying our SNRCM method enable the rapid identification of clones carrying complete BGCs. The alignment of complete BGCs derived from assembled contigs with single Nanopore reads directly reveals the presence of clones that carry complete BGCs, thus overcoming the drawback of classical screening via PCR, which can lead to the recovery of false positive clones, i.e., with a truncated rather than complete BGC. Only BGCs that do not exceed the insert size of 40 kb can be recovered from a single fosmid using our SNRCM approach; however, small BGCs such as RiPPs can nonetheless encode for therapeutically relevant compounds including antibiotics (Waisvisz et al. 1957; Schmidt et al. 2005; Scholz et al. 2011). As shown in our study, the isolation of HMW metagenomic DNA combined with short- and long-read sequencing also enables the direct recovery of complete BGCs from metagenomes via PCR and cloning into expression vectors, although direct amplification is also limited to small BGCs. For lasso peptide encoding BGCs, either of the two approaches would allow the identification and selection of those that encode an ABC transporter (D gene), as it has been observed that lasso peptides with antimicrobial activity often contain ABC transporter genes within their BGCs that confer self-resistance (Hegemann et al. 2021). With respect to the heterologous expression attempts of our amplified and cloned lasso peptide BGC 44.1, we could observe transcripts for all genes in *E. coli* (data not shown) but failed to detect the respective lasso peptide via standard high-performance liquid chromatography-mass spectrometry (HPLC–MS). In future studies, further efforts will be required to optimize compound detection and analytical methods. It is worth noting that we only covered a fraction of the metagenome because of the limiting sequencing depth and also a comparatively low library size. Nevertheless, we could prioritize among a large assortment of BGCs, and upscaling of the library size and sequencing depth is expected to lead to a greater number of complete and potentially interesting BGCs.

With respect to the recovery of BGCs spanning over multiple fosmids, our hybrid sequencing approach also proved more rapid and efficient than previous strategies. The latter made use of degenerate primers for conserved regions of biosynthetic domains to screen the library for clones of interest. Subsequently, the recovery of clones with overlapping parts was performed in order to assemble complete BGCs (Hover et al. 2018; Wu et al. 2019; Stevenson et al. 2021). These procedures require the laborious generation of a saturating genetic library with millions of clones to ensure that a chosen BGC of interest is completely covered within the library. In contrast, our approach requires comparatively smaller library sizes as the generated sequencing information immediately pinpoints BGCs that are completely covered within the library. As we have shown, sequencing data of the corresponding metagenome can serve as a reference sequence for the BGCs to be recovered. Additionally, these sequencing data can also be useful for amplifying parts of the BGC that are not covered in the corresponding library to assemble a complete cluster. Also here, upscaling of the clone number and sequencing depth can yield a greater variety of BGCs to be prioritized for recovery and subsequent heterologous expression.

The strategy described here not only enables the fast and efficient identification and recovery of a greater number of complete BGCs from a given soil metagenome and its respective genetic library, but it also expands the options for heterologous expression. In case of completely captured BGCs on fosmids such as RiPPs, the respective clones can directly be used for heterologous expression in *E. coli*. Fosmids can also be transferred into *E. coli* strains that are more suitable for heterologous expression of natural products such as phosphopantetheinyl transferase (PPtase) carrying *E. coli* strains (Gruenewald et al. 2004; Jaitzig et al. 2014). Additionally, the fosmid vector backbone can be genetically modified to enable transfer and maintenance of the plasmid in other heterologous hosts. As shown in this study, the amplification of BGCs from metagenomes allows the attachment of different restriction sites for subsequent cloning into suitable expression vectors for different hosts. Finally, different TAR vectors can be chosen for the assembly of large BGCs of interest in order to express them in different hosts such as actinobacterial, proteobacterial, or *B. subtilis* host organisms (Zhang et al. 2019). With an increasing number of recovered metagenomic BGCs, future efforts can be directed to identify more suitable hosts for heterologous expression such as species of the *Acidobacteria* phylum, which is one of the most abundant phyla in soil (Giguere et al. 2021). Alternatively, metagenomic BGCs can be codon optimized for already well-established heterologous hosts. These techniques can subsequently be used to improve actual expression rates, which still remain one of the major bottlenecks in compound production from metagenomes.

In summary, our study contributes to accelerate the discovery of new natural products by providing an approach that speeds up and facilitates the recovery of BGCs from soil metagenomes and their prompt expression in heterologous systems.

## Supplementary Information

Below is the link to the electronic supplementary material.

Supplementary file1 (XLSX 21 KB)

## Data Availability

The fosmid library sequencing data generated in this study was submitted to the NCBI Sequence Read Archive (SRA) and is accessible under BioProject identifier PRJNA799808. antiSMASH results and Nanopore read alignments are available for download from https://doi.org/10.5281/zenodo.5898572. All further relevant data generated or analyzed during this study are included in this published article and its supplementary files.
